# Tryptophan-Rich and Proline-Rich Antimicrobial Peptides

**DOI:** 10.3390/molecules23040815

**Published:** 2018-04-02

**Authors:** Awdhesh Kumar Mishra, Jaehyuk Choi, Eunpyo Moon, Kwang-Hyun Baek

**Affiliations:** 1 Department of Biotechnology, Yeungnam University, Gyeongsan, Gyeongbuk 38541, Korea; awadhesh.biotech07@gmail.com (A.K.M.); stanza15@naver.com (J.C.); 2 Department of Biological Science, Ajou University, Suwon 442-749, Korea; emoon@ajou.ac.kr

**Keywords:** antimicrobial peptide, proline-rich antimicrobial peptides, tryptophan-rich antimicrobial peptides, synthetic combinatorial libraries, membrane lysis

## Abstract

Due to the increasing emergence of drug-resistant pathogenic microorganisms, there is a world-wide quest to develop new-generation antibiotics. Antimicrobial peptides (AMPs) are small peptides with a broad spectrum of antibiotic activities against bacteria, fungi, protozoa, viruses and sometimes exhibit cytotoxic activity toward cancer cells. As a part of the native host defense system, most AMPs target the membrane integrity of the microorganism, leading to cell death by lysis. These membrane lytic effects are often toxic to mammalian cells and restrict their systemic application. However, AMPs containing predominantly either tryptophan or proline can kill microorganisms by targeting intracellular pathways and are therefore a promising source of next-generation antibiotics. A minimum length of six amino acids is required for high antimicrobial activity in tryptophan-rich AMPs and the position of these residues also affects their antimicrobial activity. The aromatic side chain of tryptophan is able to rapidly form hydrogen bonds with membrane bilayer components. Proline-rich AMPs interact with the 70S ribosome and disrupt protein synthesis. In addition, they can also target the heat shock protein in target pathogens, and consequently lead to protein misfolding. In this review, we will focus on describing the structures, sources, and mechanisms of action of the aforementioned AMPs.

## 1. Introduction

The growing level of resistance toward currently used antibiotics is a serious threat to public health, as well as to the agricultural industry. Many patients in hospitals world-wide are currently suffering from superbugs such as vancomycin-resistant enterococci, methicillin-resistant *Staphylococcus aureus* (MRSA), and drug-resistant tuberculosis [1]. In plants, copper-based derivatives and antibiotics mainly control diseases. However, the use of these compounds is limited due to the emergence of resistant strains, regulatory constraints, and environmental and health concerns [2]. Currently, antimicrobial peptides (AMPs) are increasingly viewed as an alternative to conventional antibiotics, and their modes of action are actively being elucidated [3,4]. AMPs are oligopeptides containing a varying number of amino acids (usually 8–50 amino acids, ranging in size from 2–10 kDa), typically comprising around 40% hydrophobic amino acids, and display amphipathic properties. AMPs have been isolated from both prokaryotes (e.g., bacteria) and eukaryotes (e.g., protozoan, fungi, plants, insects, and animals) [5], and are considered to be multifunctional effectors for natural defense in living organisms. Generally, they have a positive net charge ranging from +2 to +9, due to an abundance of lysine and arginine residues, which are crucial for the electrostatic interaction with negatively-charged biological membranes [6,7,8,9]. Often, due to the presence of disulfide bridges or contact with membranes, they adopt three-dimensional amphiphilic structures in which the positively charged hydrophilic domains are well separated from the hydrophobic domain. Such a combination is well suited for interacting with membranes, especially bacterial membranes with their negatively charged hydrophilic head groups and hydrophobic cores. Since cationic amino acid residues are abundant in many AMPs, they are often referred to as cationic host defense peptides (HDPs) or cationic antimicrobial peptides (CAMPs) [10]. However, some CAMPs have a low net positive charge, ranging from 0 to +3, such as temporins and bombinins H [11]. Additionally, anionic antimicrobial peptides (AAMPs) with a net charge of −1 to −7 have also been observed [12]. For example, Maximin-H5 [13] from frog skin and dermicidin [14] secreted from sweat gland tissues in humans, are anionic peptides. Moreover, AMPs have a high content of specific amino acids such as proline [15], tryptophan [16,17], glycine [18,19], cysteine [20], or histidine [21]. These variations in amino acid composition, length, and net charge are responsible for the diverse modes of action of AMPs. Among these different types of AMPs, Pro-rich AMPs (PrAMPs), and Trp-rich AMPs (TrAMPs) have attracted particular attention due to their wide distribution among many organisms, as well as they can target a number of different intracellular targets. In this review, we discuss the structure, sources, and modes of action of TrAMPs and PrAMPs.

## 2. Classification, Diversity and Mechanisms of Action of AMPs

### 2.1. Classification

Most AMPs reported to date can be classified into four groups based on their secondary structure: α-helical peptides, β-sheet peptides, extended peptides, and loop peptides [22,23]. Among these, α-helical peptides are the most studied AMPs to date and include well-established structural AMPs such as magainin [24], protegrin [25], and LL-37 [26]. A distinct lack of cysteine residues is characteristic of peptides that belong to this group. This group of peptides usually adopts a disordered structure in aqueous solution but form amphipathic helices in membranes or membrane-mimicking environments. The β-sheet containing peptides are composed of at least two β-strands with 2–4 disulfide bonds between strands and form relatively rigid structures [27]. This group includes α and β-defensins (found in many vertebrates) [28], drosomycin (found in fruit flies [29], thionins (isolated from cereals) [30], and plectasin (fungal defensin) [31]. Most α-helical and β-sheet AMPs exert their antimicrobial activities by disrupting bacterial membranes. The extended AMPs, which are predominantly rich in specific amino acids such as proline, tryptophan, arginine, glycine, and histidine, have no regular secondary structure. They may be cationic or anionic based on their net charge. The Pro-rich AMPs are exemplified by apidaecin (from bees and wasps) [32] and pyrrhocoricin (from firebeetles) [33], while the Trp-rich peptides include indolicidin [34], tritrpticin [35], and the wheat puroindoline-based peptides [36]. Histatin-5 is a histidine-rich cationic peptide present in the salivary secretions of human submandibular and parotid glands that possesses potent antifungal activity [21]. Some AMPs rich in proline and arginine cannot form amphipathic structures and may adopt β chain type polyproline or cyclic helix, for example, Nisin and PR-39. It is easily proteolytically stable and presents the considerable potential to combat emerging infectious diseases [37,38]. Many extended AMPs are not active against the membranes of pathogens, but they can achieve their antimicrobial activities by penetrating across membranes and interacting with intracellular targets. On the other hand, only a few extended peptides, such as indolicidin, are membrane active and can induce membrane leakage. The loop AMPs, including bactenecin [39] and lactoferricin (Lfcin) [40], adopt a loop formation with one intramolecular disulfide bridge.

### 2.2. Cyclic Antimicrobial Peptides

Some of the natural antimicrobial peptides with cyclic structure did not fit any of the aforementioned groups and are considered as cyclic AMP. For instance, θ-defensins possesses cyclic octadecamers and consist of a couple of antiparallel β-sheets linked by three disulfide bonds to produce a very stable structure. They are active against several gram-positive and gram-negative bacteria, fungi, and some viruses [41]. Some bacteriocins, which are polypeptide toxins produced by bacteria to inhibit the growth of competing bacterial strain (s) or species, are also cyclic AMP [42]. Tryptophan and arginine-rich cyclic hexapeptides, cyclo-RRRWFW show antibacterial activities as well as hemolytic activity [43]. Cyclotides is another type of plant-derived cyclic AMP and characterized by a head-to-tail cyclic backbone. They comprise three disulfide bonds forming the so-called cyclic cysteine knot, hence also known as “knotted peptides” [44]. There are many cyclic peptides such as polymyxin B, Gramicidin S, daptomycin, surfactin, and iturin that are also attracting significant attention. Among these, Gramicidin S and polymyxin B are two Bacilli derived cyclic peptides. Gramicidin S is a cyclodecapeptide, originally derived from gramicidin that serves as a model template for the synthesis of novel cyclic antimicrobial peptides. They exhibit strong antibacterial and antifungal activities [45]. Polymyxin B shows antibacterial activity against multi-drug resistant gram-negative bacteria and is used to treat urinary tract infections and meningitis caused by *Pseudomonas aeruginosa* and *Haemophilus influenzae*, respectively [46]. Besides, cyclotides are highly stable, retaining their biological activity after boiling and being extremely resistant to enzymatic degradation. BPC194 [c(KKLKKFKKLQ)], is a synthetic cyclic peptide and exhibited biological activity against various plant pathogens such as *Erwinia amylovora*, *Pseudomonas syringae,* and *Xanthomonas vesicatoria* [47]. Besides, cyclic dipeptides (also known as 2, 5-diketopiperazines) are mainly synthesized by microorganisms and possess diverse biological properties such as antitumor, antifungal, and antibacterial activities. For instance, bicyclomycin, Brevianamide S, and albonoursin are some well-known cyclic dipeptides [48]. Several cyclic peptides have been approved by the Food and Drug Administration (FDA) in last few years such as polymyxin B, Dalbavancin and Oritavancin. Dalbavancin and Oritavancin are useful for the treatment of skin related infection [49].

### 2.3. Diversity and Distribution

Over the last decade, more than 2800 natural antimicrobial peptides have been isolated from all six kingdoms, and more than 1000 AMPs have been reported in amphibians (Antimicrobial Peptide Database (APD); http://aps.unmc.edu/AP/main.php [50]) and their broad-spectrum activities have been reported against various pathogens including bacteria, fungi, virus, protozoa and some also with the potential for cancer therapy [51]. In animals, AMPs are mostly found in the tissues and organs that are exposed to airborne pathogens and are believed to be the first line of the innate immune defense system [52,53]. Some well-known AMPs from diverse sources are listed in Table 1.

Antimicrobial peptides are so diverse that it is difficult to categorize them, except broadly on the basis of their secondary structure. A few well documented examples of antimicrobial peptides belong to the families of the cathelicidins and defensins (found in many insects, plants and animals, including humans), thionins (isolated from plants), cecropins (found in the hemolymph of the cecropia silk moth), and magainins (secreted from frog skin) [64]. The fundamental structural principle underlying all classes of AMPs is the ability of the molecules to adopt a shape in which clusters of hydrophobic and cationic amino acids are spatially organized in discrete sectors of the molecule (i.e., an amphipathic design). Linear peptides, such as the silk moth’s cecropin A, KWKLFKKIEKVGQNIRDGIIKAGPAVAVVGQATQIAK-NH_2_ [65,66] and the African clawed frog’s magainin, GIGKFLHSAKKFGKAFVGEIMNS [24], adopt this organization only when they enter a membrane, where they assume an amphipathic α-helical secondary structure [67]. Magainin has broad-spectrum activity against bacteria, fungi, virus, protozoa as well as cancer cells. AMP such as defensins [68] use a relatively rigid anti-parallel β-sheet constrained by disulfide bonds as the framework, around which segregated patches of cationic and hydrophobic residues are organized. A large family of linear peptides characterized by a predominance of specific amino acid, such as the Trp-rich indolicidin from cow neutrophils [69] and the Pro-rich PR39 from pig neutrophils [70], segregate the hydrophobic and hydrophilic side chains around an extended peptide scaffold in the setting of the membrane. Most multicellular organisms express a cocktail comprised of multiple peptides from several of these structural classes within their defensive tissues.

There are two large families of HDPs, including cathelicidins and defensins, which have been identified in humans and other mammalian species such as cattle, pigs, sheep, and goats. Cathelicidins are small, cationic, antimicrobial peptides that are stored as inactive pro-forms, mainly in the peroxidase-negative granules of neutrophils, and also in epithelial cells and macrophages [71,72,73]. They have a well-conserved pro-peptide N-terminal segment, known as the cathelin domain, and a C-terminal domain, which is responsible for the antimicrobial properties. Only after proteolytic cleavage of the proprotein into the cathelin domain and cathelicidins is the active form released [72,74]. These proteolytically activated peptides are part of the innate immune system in many vertebrates, and are found in the lysosomes of macrophages and polymorphonuclear leukocytes. These peptides show a broad spectrum of antimicrobial activity against bacteria, enveloped viruses, and fungi. Apart from exerting direct antimicrobial effects, cathelicidins can also trigger specific defense responses in the host. Members of this family include linear peptides, among them a number of Pro-rich AMPs such as bactenecin5 (Bac5), bactenecin7 (Bac7), PR-39, and the Trp-rich indolicidin. In humans, LL-37, LLGDFFRKSKEKIGKEFKIVQRIKDFLRNLVPRTES is the only member of the cathelicidin family [26,75]. The precursor protein is referred to as hCAP18 (human Cationic Antibacterial Protein of 18 kDa). The protein is cleaved by serine proteases to release a peptide containing 37 amino acids beginning with two leucines, from the C-terminal domain and hence the name LL-37. Serine proteases released from keratinocytes such as kallicreins and neutrophil proteases [76,77]. The minimum inhibitory concentrations (MIC) of LL-37 against *Escherichia coli* and *Staphylococcus aureus* (~64 µM) while against *Candida albicans* (20 µM) were found [78].

Defensins, first discovered in human neutrophils, are cysteine-rich, 18-45 amino acid long cationic molecules, which have been isolated from mast cells and tissues and are involved in host defense [68]. These include three families, α-, β-, and θ-defensins that are rich in cysteine residues and are classified according to the arrangement of their disulfide bridges [28,79]. α- and β-defensins are widely distributed in vertebrates, predominantly, in phagocytes (neutrophils and macrophages), small intestinal Paneth cells, and mucosal epithelial surfaces in various organs [80]. θ-defensins, the only known macrocyclic AMPs of animal origin, are expressed in the leukocytes of rhesus macaques and olive baboons [81]. Some peptides are derived by proteolysis from larger proteins, such as buforin II (TRSSRAGLQFPVGRVHRLLRK) from histone 2A [82] and Lfcin (FKCRRWQWRMKKLGAPSTTCVRRAF) derived from the N-terminus of iron-binding lactoferrin protein [83].

Why does diversity arise? Because changes in a single residue can dramatically alter the biological activity of each peptide, AMP diversity probably reflects the species adaptation to the unique microbial environments that characterize the niche occupied, including the microbes associated with acceptable food sources [84,85]. It seems reasonable to speculate that an individual could find itself in the midst of microbes against which its antimicrobial peptides are ineffective; although the individual might suffer, the species itself could survive through the emergence of individuals expressing beneficial mutations. Adaptive immunity, through its plasticity, permits a species to empower individuals to explore new environments and avail themselves of new food sources. Natural peptides generally composed of d-amino acids, in place of l-amino acids, typically retain full antibiotic potency while exhibiting the expected resistance to enzymatic proteolysis. Few short linear or cyclic peptides contain both l- and d-amino acids as well as non-proteinogenic amino acid, can be generated with various degrees of selectivity and antimicrobial potency [86]. Understanding the structure-activity relationships of AMPs is essential for the design and development of novel antimicrobial agents with improved properties. In particular, the atomic level structures of AMPs can provide useful information for all stages of drug development, including peptide design and modifications for pharmaceutical applications. The α-helical magainins, which are isolated from the skin of *Xenopus laevis* (African clawed frog), represent the best-characterized amphibian AMPs [24,87]. Pexiganan (also known as MSI-78), a synthetic analog of magainin 2, is one of the most studied AMPs in terms of drug development. Locilex^®^ (pexiganan cream 0.8%, PLx Pharma Inc., Houston, TX, USA), is antibiotic for the treatment of moderate diabetic foot infections and currently in Phase 3 clinical trials. Two omiganan (an indolicidin analog)-based AMPs, MBI-226, and MX-594AN, have also been developed for the treatment of catheter-related infections and acne, respectively [88,89]. These two omiganan-based drugs have also successfully completed phase 3 clinical trials (https://clinicaltrials.gov/ct2/show/NCT02576847).

### 2.4. Mechanisms of Action

AMPs show a broad spectrum of antimicrobial activities against various microorganisms. They can kill cells either by disrupting membrane integrity [7], by inhibiting macromolecule synthesis (proteins, DNA and RNA), or by targeting metabolic enzymes [4]. Bacterial membranes are organized in such a way that the outermost leaflet of the bilayer (i.e., the surface exposed to the outer world) is heavily populated by lipids with negatively charged phospholipid head groups [10,22]. In contrast, the outer leaflet of the membranes of plants and animals is composed principally of lipids with no net charge; most of the lipids with negatively charged head groups are segregated into the inner leaflet, facing the cytoplasm [10]. A model that explains the activity of most antimicrobial peptides is the Shai-Matsuzaki-Huang (SMH) model [22,90]. The model describes the interaction of the peptide with the membrane, followed by displacement of lipids, alteration of membrane structure, and in certain cases entry of the peptide into the interior of the target cell. In general, peptides operating by the SMH mechanism kill microbes at micromolar concentrations. In contrast, the antibiotic peptide Nisin, a 14-amino-acid amphipathic molecule produced by *Lactococcus lactis*, is an exception since it is active at nanomolar concentrations. Nisin is a bacteriocin that binds with high affinity to Lipid II, the fatty acyl proteoglycan anchor in the bacterial membrane, from which it subsequently diffuses into the surrounding membrane [37,91]. In some cases, AMPs have been shown to kill antibiotic-resistant bacteria. For example, both nisin (an AMP) and vancomycin (an antibiotic) can block cell wall synthesis. However, a methicillin-resistant *S. aureus* (MRSA) strain was reported to be resistant to vancomycin, whereas it was still sensitive to Nisin [92].

This antibacterial activity is attributed to non-receptor-mediated membrane-lysis which directly interferes with the integrity of the bacterial cell membrane. It has been postulated that membrane-active AMPs selectively disrupt the cell membrane to form pores that allow for the efflux of essential ions or nutrients [22,90]. Based on the SMH model, most AMPs act via an interaction with the membrane resulting in a morphological change in membrane structure [90,93]. Various models of pore formation have been proposed and discussed in several reviews [5,79,93,94]. These include three types of disruption method, namely, the toroidal-pore, barrel-stave, and carpet models. In the toroidal-pore model, initial binding of the peptide to the membrane is followed by cascade aggregation of incoming monomer units, causing the lipid moieties of the outer and inner membranes to fold inward, forming a continuous channel lined by multiple peptide units [95]. The lipid head groups of membrane phospholipids are thus tightly associated with the peptides. Examples include melittin [96], magainin 2 [97], and lacticin Q [98]. The barrel-stave model differs from the toroidal-pore model in that the peptide monomers inserted into the membrane are arranged parallel to the phospholipid molecules of the membrane. The lumen of the transmembrane channel is lined by the hydrophilic side of the peptides, while the hydrophobic side faces outwards in association with the lipid core of the bilayers [95]. An example of an AMP of this type is alamethicin [96]. In the carpet model, the cumulative effect of peptides interacting with the outer membrane induces a local weakness, causing a disintegration of the membrane structure and giving a carpet-like appearance [95]. An example of a carpet model AMP is cecropin P1 (found in pig roundworm) [99].

In recent years, there has been increasing evidence that intracellular targeting AMPs can kill microbes without causing membrane damage [15,94,100]. These intracellular targeting mechanisms can be divided into several groups, including inhibition of macromolecular (nucleic acid or protein) synthesis [101], inhibition of metabolic/enzymatic function [102], and inhibition of cell-wall/membrane formation [103]. For example, buforin II (from the Chinese toad *Bufo gargarizans*), can diffuse into cells and bind to DNA and RNA without damaging the cell membrane [104]. Drosocin, pyrrhocoricin, and apidaecin are other examples of such AMPs that inhibit protein synthesis and refolding [105,106]. AMPs acting through intracellular targets are able to traverse the cell barriers to reach intracellular targets. The uptake process of AMPs generally occurs via direct penetration or endocytosis [107]. Direct penetration is energy independent pathways that include different above-described mechanisms such as pore formation and carpet-like model. Endocytosis follows either macropinocytosis or receptor-mediated pathways [108]. Macropinocytosis is an energy-dependent uptake mechanism that facilitates the formation of AMP-carrying macropinosomes derived from folding of the target cell membrane [107]. In the receptor-mediated pathway, the formation of small vesicles in the cytoplasm is preceded by pit formation mediated by clathrin or caveolin membrane proteins [108,109]. Thus, the initial interaction between the peptides and the microbial cell membrane would allow them to penetrate into the cell to bind intracellular molecules, resulting in the inhibition of cell wall biosynthesis and DNA, RNA, and protein synthesis.

## 3. Screening of Short Synthetic AMPs Using a Synthetic Combinatorial Library (SCLs) Approach

Although native AMPs have many advantages, practical application has been delayed because of high MICs, high cytotoxicity to animal cells, limited natural sources from which they can be purified, as well as high synthetic cost [110]. Furthermore, the use of natural AMPs is often limited due to a low level of activity and/or poor bioavailability [111]. As an alternative, a positional scanning approach using mixture-based synthetic combinatorial libraries (SCLs) was introduced in the 1990s [112]. An SCL is comprised of tens of millions of peptide sequences, and positional scanning of such libraries has enabled the successful identification of antimicrobial peptides with new or novel properties in a short period of time [113,114,115]. Three different types of libraries are currently available; phage display, a solid support bounded library, and a soluble library. Among them, only the soluble form of the library has been successfully used to identify new types of antimicrobial peptides [113,114]. The primary advantage of using soluble libraries is the ability to screen compounds that are in solution against a cell suspension in a simple and rapid way. For example, Houghten et al. [113] were able to identify highly active individual compounds from a library of 52 million compounds within two weeks.

In addition, the SCL approach has been used to improve the activity of existing antimicrobial amphipathic peptides. A ten-fold enhancement in activity was obtained during a screening of a SCL built amphipathic 18-mer peptide, with a sequence already known to have antimicrobial activity [116]. Many AMP antibiotics have been selected using the SCL approach over the last decade, most of which were targeted toward human pathogens. SCL approaches have also been used to target plant pathogens and most AMP selection approaches have been successfully designed against fungal pathogens [117]. Given the limits of currently available AMPs, small synthetic antimicrobial peptides (ssAMPs; typically less than 10 amino acids) could be good alternatives since the cost of synthesis is much lower than that of long native AMPs. The sequences of ssAMPs can be selected by a quick screening of synthetic oligopeptide libraries [118], or through logical design based on available data, while still retaining most of the features of native AMPs. The designed ssAMPs have been studied intensively in the pharmaceutical sector. Synthetic short peptides containing R and W repeats have shown that tetrapeptides are sufficient to kill bacteria, regardless of the amino acid order [119]. When the effects of chain length on the activities of AMPs with repeating (RW)*n*-NH_2_ units (*n* = 1, 2, 3, 4 or 5) were analyzed, hexapeptides (RW)_3_-NH_2_ were found to be the optimal chain length, since although the longer chains had more antibacterial potential they also increased the hemolysis of red blood cells [120]. Selecting new ssAMPs using positional scanning of a synthetic peptide combinatorial library (PS-SPCL) can provide a more diverse pool of potential AMPs than a simple logical design approach [111,121,122]. In our previous study, four novel hexapeptides were selected through rapid screening of a synthetic combinatorial library [122] and among them, the hexapeptide KCM21 had a more effective antimicrobial activity. Recently, we have also constructed another PS-SPCLs. In particular, the hexapeptide named BHC10 (KWWDRF) has antimicrobial activity against wild-type as well as three streptomycin-resistant mutants of *Xanthomnas citri* subsp. *citri* [123]. Moreover, L-amino acid containing natural peptides are not considered to be effective therapeutic agents, owing to their lack of oral bioavailability, and their rapid enzymatic degradation, although a number of peptide antibiotics have been shown to be of value as intravenous and topical therapeutics (e.g., bacitracin). The versatility of SCLs easily overcomes these problems by introducing the d-form enantiomer, as well as unnatural amino acids, into the peptide SCLs. Thus, PS-SPCL can therefore, provide a rich pool for synthesizing small AMPs at low cost, and provide a major advance in the discovery of new antimicrobial peptides to control human as well as plant diseases [121,124,125,126].

## 4. Trp-Rich AMPs (TrAMPs)

Trp residues are found in high proportion (more than 25%) in a specific subset of antimicrobial peptides with sizes generally ranging from 5 to 11 residues [119,127]. The Trp residues appear to prefer to position specifically near the C-terminal end [128]. Furthermore, many Trp-rich AMPs cross bacterial membranes without affecting membrane integrity and act via intracellular targets. Only a few have been shown to act via a membrane lysis mechanism, e.g., Indolicidin. TrAMPs are often found to also contain arginine residues. Such peptides thus display activity against a range of bacteria and fungi (summarized in Table 2). Others are also active against viruses and cancer cells e.g., indolicidin and bovine lactoferricin (LfcinB) (discussed below). 

### Significance of Trp Residues and Mechanism of Action

Trp is an aromatic, neutral amino acid, and is the largest amino acid containing its characteristic indole functional group. Trp can sit at different positions on the hydrophobicity scale; e.g., Trp can be considered hydrophobic due to its uncharged side chain. However, Trp side chain is not embedded in the hydrocarbon region of lipid bilayers and positions itself towards the hydrophilic side [129]. These hydrophilic and hydrophobic attributes of Trp make it ideal for insertion into membranes [130]. It has a strong tendency to insert itself into membranes and partitions near the membrane-water interface, preferring to locate specifically near the lipid-carbonyl region [128,131]. Due to its aromatic side chain, Trp is able to rapidly form hydrogen bonds with the bilayer components, having a dipole moment of ~2.1 debyes [132]. Indole side chains Trp possesses an intrinsic preference for a specific orientation when positioned at the interface of a phospholipid bilayer [16]. The large, paddle-like, indole side-chain in Trp, which is about a third of the thickness of a phospholipid monolayer, interrupts the cohesive hydrophobic interactions of the lipid acyl chains when this bulky side chain buries itself deep into the hydrocarbon core of the bilayer. TrAMPs are often found to contain arginine (Arg) residues which have a positively charged guanidinium group located at the end of the side chain. The positively charged guanidinium help in the first step of attracting the TrAMPs to target membranes by forming hydrogen bonds (H-bonds) with the negatively charged membrane components [133]. Additionally, the guanidinium head group is pivotal for the uptake of cell-penetrating peptides, as it also allows for both electrostatic and H-bonding with anionic and polar molecules. These unique properties make the Arg- and Trp-rich AMPs highly active even at very short peptide lengths [119]. The significance of these two amino acids for antimicrobial activity has been highlighted by Combi-1 and Combi-2 [111]. Additionally, these two residues are capable of participating in cation–π interactions (between the indole ring of Trp and the guanidium group of Arg), thereby facilitating enhanced peptide-membrane interactions. This interaction has been observed for example in the antimicrobial peptide derived from human lactoferrin [134].

The special features of the Trp residue are useful in designing short peptide antibiotics [135]. To elucidate the main structural requirement for such short AMPs, a large number of these peptides have been synthesized [136,137]. It has been noted that a minimum of two Trp residues with amidated C-terminal end, are essential for the antimicrobial activity of short synthetic peptides [122,138,139]. Furthermore, a minimum size of six amino acid residues is required for high antimicrobial activity against the gram-negative bacteria *E. coli* and the gram-positive bacterium *S. aureus*. The position of the Trp residues, rather than their number in AMP, greatly influence antibacterial activity [122,139]. The insertion of additional Trp residues into the peptide results only in small variations in antimicrobial activity, whereas replacement of a Trp residue with tyrosine or alanine in hepta- and hexapeptides results in reduced antimicrobial activity, especially against gram-negative bacteria. Another synthetic hexapeptide PEP6 (FRLKFH) is a with activity against *Fusarium oxysporum* f. sp. *lycopersici*, *Rhizoctonia solani*, *Ceratocystis fagacearum*, and *Pythium ultimum* [140]. Another synthetic antifungal hexapeptide, PAF26 is active against *Penicillium italicum*, *P. digitatum*, and *Botrytis cinerea* [117]. The antibacterial active fragment of LfcinB, the hexapeptide RRWQWR, was identified by Tomita et al. in 1994 [141]. Moreover, in a previous study using the approach of positional scanning of a synthetic peptide combinational library, four novel hexapeptides were discovered and shown to have antimicrobial activity; KCM11: TWWRWW-NH_2_, KCM12: KWRWIW-NH_2_, KCM21: KWWWRW-NH_2_, and KRS22: WRWFIH-NH_2_ [122]. Their bactericidal activity was demonstrated using a clear zone assay against phytopathogenic bacteria including, *Pectobacterium* spp., *Xanthomonas* spp., and *Pseudomonas* spp. as well as others. Trp-rich AMPs have also been examined for their antiviral activity. Indolicidin ((ILPWKWPWWPWRR-NH_2_) isolated from bovine neutrophils most extensively characterized TrAMP. They exhibit a broad range of activities against bacteria, fungi, protozoa, and virus (discussed in Table 2) [101]. Besides, they have been also useful in the treatment of MRSA infection. Indolicidin and its synthetic analogue R12-OH (ILPWKWPWWPWR), have been used as antiviral agents against the enveloped human immunodeficiency virus (HIV)-1 by inhibiting integrase [142]. Indolicidin also exhibits a strong activity against herpes simplex virus, directly inactivating viral particles without cytotoxicity in a Vero cell line [143]. Another important TrAMP, Tritrpticin (VRRFPWWWPFLRR) is derived from a precursor protein in the porcine bone marrow and high homology to indolicidin. They possess potent bactericidal and fungicidal activity against several clinically microorganism [35]. Lactoferricin are mammalian TrAMP derived from lactoferrin and exhibit antimicrobial activity against bacteria, fungi as well viruses [83]. In all lactoferricin, a loop region with an intramolecular disulfide bridge is present and structure of disulfide bond is differing among species to species. Further, antimicrobial activities of lactoferricin differ from species to species. For example, the MIC of bovine Lfcin (FKCRRWQWRMKKLGAPSTTCVRRAF) against certain *E. coli* strains was found to be ~30 µg/mL whereas a MIC of with 100 µg/mL was detected for human Lfcin (TKCFQWQRNMRKVRGPPVSCIKRDS) [144]. Bovine Lfcin has been shown to possess antiviral activity against human cytomegalovirus [145] and hepatitis C virus [146]. Puroindolines are isolated from wheat grain TrAMP and contain two variants named as Puroindoline A and Puroindoline B. Both contains five disulfide bonds and have shown antibacterial and antifungal activities [36].

## 5. Pro-Rich AMPs (PrAMPs)

PrAMPs belong to the group of cationic peptides that are enriched in Pro residues and are often arranged in conserved patterns along with arginine residues. Collectively, they have low sequence homology, but are structurally homologous (about 30% Pro content, one or several Pro-Arg-Pro motifs, and a disordered structure in solution) and appear to kill bacteria by non-lytic mechanisms [159,160,161]. Several natural proline-rich cyclopolypeptides isolated from marine organisms possess potential therapeutic activities [162]. In gram-negative bacteria, the majority of PrAMPs are actively transported across the bacterial membrane into the cytoplasm by a specialized transporter-mediated uptake mechanism such as SbmA. The mutation in membrane protein SbmA showed partial resistance against many PrAMP but, the physiological role of SbmA still remains unclear [163,164]. The first intracellular AMP target identified was the heat-shock protein DnaK [102,164,165]. DnaK is the major bacterial heat shock protein 70 and represents an important component in *E. coli* chaperone pathways [166]. However, the findings that a DnaK null mutant retained susceptibility to PrAMPs suggests that there are other targets [105,167]. Recent reports have shown that PrAMPs bind to the 70S ribosome as the main target and interfere with the process of protein synthesis [105,106,168]. PrAMP interaction with the 70S ribosome is facilitated by a multitude of hydrogen bonds and stacking interactions with the help of cationic and hydrophobic amino acids. However, sequence diversity in the N-terminal domain in PrAMPs leads to different types of interactions with the ribosome. Oncocin and Bac7_1–35_ (a C-terminal truncated form of Bac7) inhibits protein synthesis at the initiation stage by binding to the peptide exit tunnel [168]. Hence, PrAMPs kill the microbial pathogens via intracellular targets, without disrupting membrane integrity. These features make them attractive for both basic and applied research efforts and provide novel insights into the mechanism of action of anti-infective chemical agents in both bacterial and eukaryotic cells.

PrAMPs appear to be expressed in some arthropods (insects and crustaceans), as well as in animals (summarized in Table 3). The first PrAMP, apidaecin, was, discovered in honey bees in late 1980. The discovery of apidaecin was quickly followed by the identification of other insect and mammalian PrAMPs. PrAMPs include abaecin from the honey bee (*Apis mellifera*) [169], drosocin from the fruit fly (*Drosophila melanogaster*) [170], pyrrhocoricin from the firebeetle (*Pyrrhocoris apterus*) [33], metalnikowin-1 from the green shield bug (*Palomena prasina*) [171], and oncocin from the milkweed bug (*Oncopeltus fasciatus*) [172]. The PrAMP Arasin1 has been isolated from the spider crab (*Hyas araneus)* [173]. Riptocin is another PrAMP isolated from the midgut of bean bug (*Riptortus pedestris)* that showed high antimicrobial activity against *E. coli* and *S. aureus* [174,175]. Bactenecin is a mammalian PrAMP that has been identified in ruminant species (cows, sheep, and goats) and is referred to as Bac5 or Bac7 depending on the molecular weight of the mature peptide [176]. In pigs, the corresponding PrAMP homolog to Bac7 is referred to as PR-39, due to its length of 39 amino acids [70,177]. The PrAMPs can be further divided into short chain (20 residues or fewer) and long-chain (more than 20 residues) subfamilies, the former demonstrating more potent activity against gram-negative bacteria, whereas the latter is more active against gram-positive bacteria and fungi [178,179,180].

All mammalian PrAMPs belong to the cathelicidin group of host defense effectors, with the defensins representing the two most widespread families of vertebrate host defense peptides. Similar to non-Pro-rich mammalian AMPs, Bac5/Bac7 peptides are produced by immature myeloid cells as pre-pro-peptide precursors. The Bac5 and Bac7 pre-pro peptides comprise a 29 amino acid pre-signal followed by a 101 amino acid pro-region. Bac5/Bac7 is targeted to large granules, where the targeting signals are cleaved upon import to yield pro-peptides in differentiated neutrophils. When the immune system recognizes invading bacteria, the maturation of pro-Bacs is triggered by secretion and mixing with the contents of large azurophilic granules [70]. The mature Bac5 (43 amino acid) and Bac7 (60 amino acid) peptides can then pass through the bacterial cell membrane via the SbmA transporter, where they can subsequently interact with their intracellular target. In general, PrAMPs enter into bacteria through the inner membrane via translocation involving a transporter-mediated uptake mechanism mediated by the membrane protein SbmA [163]. Furthermore, a recent report has shown that several PrAMPs, including goncocin, apidaecin, and drosocin, can cross the blood-brain barrier to selectively target brain cells [181,182]. These data indicate that PrAMPs are not only potential therapeutics for cerebral infections, but could also be novel potential carriers for brain drug delivery.

## 6. Resistance to Antimicrobial Peptides

Resistance mechanisms to AMPs are generally divided into two different types: constitutive resistance and inducible resistance [189]. The inducible resistance mechanisms include substitution [190], modification [191], and acylation [192] of the membrane molecules, activation of proteolytic enzymes [193], transport through efflux pumps [194], and modifications of the intracellular targets [195]. The constitutive resistance mechanisms include electrostatic shielding [196], changes in membrane potential during different stages of cell growth [197], and biofilm formation [189].

For example, the activity of some AMPs against *S. aureus* can be inhibited by adhesin molecules present on the cell surface of this bacterium. These adhesin molecules are polymeric substances and stay on the cell surface after secretion [198]. Since adhesin is a positively charged polymer, it can form a repulsive barrier against positively charged AMPs. Additionally, many species of gram-negative bacteria, such as *E. coli* and *S. typhimurium*, possess a membrane-bound lipid A modification system that can help defend themselves against host-derived AMPs [199]. In this system, resistance is provided by the PhoQ/PhoP system. PhoQ is a membrane-bound sensor kinase and PhoP is the intracellular response regulator. PhoQ is activated in the presence of high-levels of Ca^2+^, Mg^2+^, or Mn^2+^ outside cells and leads to a change in the conformation of PhoQ. PhoQ then autophosphorylates and activates PhoP causing the up-regulation of genes, including those related to AMP resistance. This system is not active when the extracellular levels of these positive ions is low [200]. However, it is difficult to develop complete resistance against AMPs since each resistance mechanism only covers a few types of AMP.

## 7. Concluding Remarks

At present, AMPs represent one of the most promising future approaches for fighting microbial drug resistance. This is evident by the increasing number of studies involving these peptides. With the rapid growth in knowledge and the availability of lead compounds, more AMPs may enter clinical trials in the near future. Although AMP structure is certainly important, peptide size, charge and solubility are all crucial physicochemical properties that are also important for AMP antimicrobial activity and target specificity. Modification of any of these parameters will lead to subsequent alterations in biological activity and the target spectrum of AMPs. Using the synthetic library approach, it is easy to alter the peptide residue sequence and ultimately affect major changes in antimicrobial activity. However, predicting the results of these changes is not easy. Thus, there is a need to understand the effects of structural modifications of AMPs on their physiochemical characteristics, as well as their target spectrum and activity. Recently, these types of studies have increasingly used computational approaches. In the future, it is hoped that these approaches will help to better understand the mode of action of AMPs and predict their activities. In TrAMPs, the position of Trp, rather than the number of Trp residues, in these peptides is an important factor in their antimicrobial activity. A minimum length of six amino acids is required for complete antimicrobial efficiency in synthetic AMPs. The proline content in the primary sequence of an AMP has been found to affect its ability to penetrate cell membranes, due to its effect on secondary structure. PrAMPs inhibit protein synthesis as their common mechanism of action and predominantly sourced from insects. In addition, they also cause protein misfolding by targeting the heat shock protein DnaK. They have the capability to cross the blood-brain barrier, which may afford exciting new opportunities for drug delivery as potential drug carriers. Although, there are various advantages to TrAMPs and PrAMPS, challenges still remain to their application. These include susceptibility to proteases and extreme pH, lack of selectivity against specific strains, and high production costs. In addition, mutation of the SbmA transporter is also a major concern that could increase resistance against antimicrobial peptides. Cyclic AMP could be a more suitable alternative to peptide drugs development, since they are more resistant to enzymatic degradation and extreme pH.

## Figures and Tables

**Table 1:** AMPs and their sources.

Source of AMPs	AMPs	References
Amphibians	Japonicin-2, Nigrocin-2, Temporin, Dermaseptin, Magainin, Buforin II	[54]
Insect	Cecropin, Thanatin, Defensin, Drosomycin, Drosocin, Metchnikowin, Apidaecin, Abaecin, Pyrrhocoricin, Melittin	[55]
Crustaceans	Callinectin, Homarin, Penaeidin, Hyastatin, Arasin	[56]
Plants	Thionins, Plant Defensins	[57]
Mammals	Defensin, Histatin, LL-37, Indolicidin, Protegrin, Lactoferricin	[58,59]
Bacteria	Iturin, Bacillomycin, Syringomycin	[60]
Fungi	Echinocandins, Aculeacins, Aureobasidin	[61]
Fishes	Pardaxins, Misgurin, Pleurocidins, Parasin	[62]
Echinoderms	Strongylocins, Centrocins, Betathymosins	[63]

**Table 2:** TrAMPs and their antimicrobial activities (hexapeptide TrAMPs are in shown underlined).

Name (Source)	Peptide Amino Acid Sequence and Length	Antimicrobial Activities					References
		Gram-Negative	Gram-Positive	Fungi	Protozoa	Virus	
Indolicidin (Bovine neutrophils)	ILPWKWPWWPWRR-NH_2_ (13)	*E. coli*, *Salmonella typhimurium*, *Pseudomonas aeruginosa*	*S. aureus*, *S. epidermidis*	*C. albicans*	*Giardia lamblia*, *Eimeria acervulina*	*Tobacco Mosaic Virus*, *Human Immunodeficiency Virus*, *Herpes Simplex Virus*	[34,69,101,142]
Tritrpticin (Porcine bone marrow)	VRRFPWWWPFLRR (13)	*E. coli*, *S. typhimurium*, *P. aeruginosa*	*Bacillus subtilis*, *S. aureus*, *S. epidermidis*	*C. albicans*	N/A	N/A	[35,147,148,149]
Puroindoline A (Wheat grain)	FPVTWRWWKWWKG-NH_2_ (13)	*E. coli*	*S. aureus*	*Colletotrichum graminicola*, *R.solani*, *R. cerealis*	N/A	N/A	[36,150,151]
Puroindoline B (Wheat grain)	FPVTWPTKWWKG-NH_2_ (12)	*E. coli*	*S. aureus*	*R. solani*, *R. cerealis*	N/A	N/A	[36,150,151]
Combi-1 (Synthetic, combinatorial peptide)	Ac-RRWWRF-NH_2_ (6)	*E. coli*	*S. aureus*, *S. sanguis*	*C. albicans*	N/A	N/A	[111]
Combi-2 (Synthetic, combinatorial peptide)	Ac-FRWWHR-NH_2_ (6)	*E. coli*	*S. aureus*, *S. sanguis*	*C. albicans*	N/A	N/A	[111]
Cyclo-combi (Synthetic, combinatorial peptide)	RRWWRF (6)	*E. coli*	*S. aureus*, *S. sanguis*	*C. albicans*	N/A	N/A	[111]
KCM21 (Synthetic, combinatorial peptide)	KWWWRW-NH_2_ (6)	*Pectobacterium carotovorum*, *P. syringae* pv. *tomato* DC3000, *Xanthomonas campestris*, *X. citri* subsp. *citri*	*Clavibacter michiganensis* subsp. *michiganensis*	*C. krusei*	N/A	N/A	[122,152]
Bovine lactoferricin (Bovine lactoferrin)	FKCRRWQWRMKKLGAPSTTCVRRAF (25)	*E. coli*, *Klebsiella pneumonia*, *P. aeruginosa*, *Proteus vulgaris*, *Salmonella enteritidis*, *Yersinia enterocolitica*,	*B. cereus*, *B. subtilis*, *Clostridium perfringens*, *L. monocytogenes*, *S. aureus*, *Streptococcus bovis*, *S. epidermidis*, *Staphylococcus haemolyticus*, *Staphylococcus hominus*	*C. albicans*, *C. tropicalis*, *Cryptococcus neoformans*, *Trichophyton mentagrophytes*, *T. rubrum*	*Toxoplasma gondii*, *Eimeria stiedae*	*Human Cytomegalovirus*, *hepatitis C virus*, *Human Immunodeficiency Virus*, *Herpes Simplex Virus*	[40,153,154]
LfcinB_4-9_ (Shorter derivatives of bovine lactoferricin)	RRWQWR-NH_2_ (6)	*E. coli*	*S. aureus*	*C. albicans*	N/A	N/A	[141]
LysH (Derived from C-terminus region of human lysozyme)	RAWVAWR-NH_2_ (7)	*E. coli*, *K. pneumonia*, *P. aeruginosa*, *Serratia marcescens*	*B. subtilis*, *Micrococcus luteus*, *S. aureus*, *S. epidermidis*, *Staphylococcus lentus*, *Streptococcus zooepidemicus*	N/A	N/A	N/A	[155]
LysC (Derived from C-terminus region of egg white lysozyme)	IVSDGNGMNAWVAWR-NH_2_ (15)	*E. coli*, *K. pneumonia*, *S. marcescens*	*B. subtilis*, *S. aureus*, *S. zooepidemicus*	N/A	N/A	N/A	[155]
(RW)_3_ (Rationally designed synthetic peptide)	RWRWRW-NH_2_ (6)	*E. coli*	*S. aureus*	*Fusarium solani*, *F. oxysporum*	N/A	N/A	[120,156]
Grain softness protein (Gsp)-1 (Synthetic peptide, derived from wheat grain)	MPLSWFFPRTWGKR-NH_2_ (14)	*E. coli*	*S. aureus*	*C. graminicola*, *R. solani*	N/A	N/A	[36]
GA-K4AL (Synthetic peptide, derived from skin of Korean frog *Glandirana emeljanovi*)	FAKWAFKWLKK-NH_2_ (11)	*E. coli*, *K. pneumonia*, *S. dysenteriae*, *S. typhimurium*	*S. aureus*, *S. epidermidis*, *P. aeruginosa*, *B. subtilis*, *M. luteus*	N/A	N/A	N/A	[157]
PW2 (Synthetic peptide, isolated from phage display library)	HPLKQYWWRPSI (12)	N/A	N/A	*Eimeriatenella*, *E. acervulina*, *C. albicans*, *A. nidulans.*	N/A	N/A	[158]
PAF2 6(Synthetic, combinatorial peptide)	Ac-RKKWFW-NH_2_ (6)	N/A	N/A	*P. digitatum*, *P. italicum*, *P. expansum*, *B. cinerea*, *F. oxysporum*, *M. grisea*	N/A	N/A	[117]
PEP6 (Synthetic peptide)	FRLKFH (6)			*F. oxysporum*, *R. solani*, *C. fagacearum* *P. ultimum*			[140]

Ac: acetylation; NH_2_: amidation; Peptide without modified C- and N-terminus stands for their free end; N/A: Not available.

**Table 3:** Proline-rich AMP, their sequences and mode of action.

PrAMP (Source)	Peptide Amino Acid Sequence and Length	Mode(s) of Actions	References
Apidaecin Honey bee (*A. mellifera*)	GNNRPVYIPQPRPPHPRL (18)	Inhibits protein biosynthesis by targeting 70S ribosomes, inhibits DnaK, inhibits ABC transport system and binds to LPS	[32,105]
Abaecin Honey bee (*A. mellifera*)	YVPLPNVPQPGRRPFPTFPGQGPFNPKIKWPQ (32)	Inhibits protein biosynthesis by targeting 70S ribosomes, inhibits DnaK	[169]
Drosocin Fruit fly (*D. melanogaster*)	GKPRPYSPRPTSHPRPIRV (19)	Inhibits protein biosynthesis by targeting 70S ribosomes, inhibits DnaK and GroEL, binds to LPS	[170]
Pyrrhocoricin Fire beetle (*P. apterus*)	VDKGSYLPRPTPPRPIYNRN (20)	Inhibits protein biosynthesis by targeting 70S ribosomes, inhibits DnaK and GroEL, binds to LPS	[33,168]
Metalnikowin-1 Green shield bug (*P. prasina*)	VDKPDYRPRPRPPNM (15)	Inhibits protein biosynthesis by targeting 70S ribosomes, inhibits DnaK and GroEL, binds to LPS	[168,171]
Oncocin Milkweed bug (*O. fasciatus*)	VDKPPYLPRPRPPRRIYNR-NH_2_ (19)	Inhibits protein biosynthesis by targeting 70S ribosomes, inhibits DnaK	[105,168,172,183]
Arasin1 Spider crab (*H. araneus*)	SRWPSPGRPRPFPGRPKPIFRPRPC (25)	Inhibits protein biosynthesis by targeting 70S ribosomes, inhibits DnaK	[173]
Riptocin Bean bug *(R. pedestris)*	VDKGGYLPRPTPPRPVYRS (19)	Inhibits protein biosynthesis by targeting 70S ribosomes, inhibits DnaK	[174,175]
Penaeidins-1 Whiteleg shrimp (*Penaeus vannamei*)	YRGGYTGPIPRPPPIGRPPLRLVVCACYRLSVSDARNCCIKFGSCCHLVK (50)	Inhibits protein biosynthesis by targeting 70S ribosomes, inhibits DnaK	[184]
Bac5_1-23_ Cows (*Bos taurus*)	RFRPPIRRPPIRPPFYPPFRPPI (23)	Inhibits protein biosynthesis by targeting 70S ribosomes, inhibits DnaK	[39]
Bac7_1-35_ Cow (*Bos taurus*)	RRIRPRPPRLPRPRPRPLPFPRPGPRPIPRPLPFP (35)	Inhibits protein biosynthesis by targeting 70S ribosomes, inhibits DnaK	[39,185,186]
PR-39 Wild swine (*Sus scrofa*)	RRRPRPPYLPRPRPPPFFPPRLPPRIPPGFPPRFPPRFP (39)	Inhibits protein biosynthesis by targeting 70S ribosomes, inhibits DnaK, inhibits septation, affects coenzyme transport and metabolism, acts as a non-competitive and reversible inhibitor of the 20S proteasome	[70,187]
BnPRP1 Rapeseed (*Brassica napus)*	PPTQNPSMAPPTQNPYGQPMTPPTQNPYGQPMAPP (35)	Inhibits protein biosynthesis by targeting 70S ribosomes, inhibits DnaK	[188]

NH2: amidation; Peptide without modified C-and N- terminus stands for their free end.

## References

[1] World Health Organization (2014). Antimicrobial Resistance: Global Report on Surveillance.

[2] Sundin G.W., Bender C.L. (1993). Ecological and genetic analysis of copper and streptomycin resistance in *Pseudomonas syringae* pv. syringae. Appl. Environ. Microbiol..

[3] Kang H.K., Kim C., Seo C.H., Park Y. (2017). The therapeutic applications of antimicrobial peptides (AMPs): A patent review. J. Microbiol..

[4] Le C.-F., Fang C.-M., Sekaran S.D. (2017). Intracellular targeting mechanisms by antimicrobial peptides. Antimicrob. Agents Chemother..

[5] Reddy K.V.R., Yedery R.D., Aranha C. (2004). Antimicrobial peptides: Premises and promises. Int. J. Antimicrob. Agents.

[6] Fernández-Vidal M., Jayasinghe S., Ladokhin A.S., White S.H. (2007). Folding amphipathic helices into membranes: Amphiphilicity trumps hydrophobicity. J. Mol. Biol..

[7] Hancock R.E.W. (1997). Peptide antibiotics. Lancet.

[8] Powers J.-P.S., Hancock R.E.W. (2003). The relationship between peptide structure and antibacterial activity. Peptides.

[9] Hancock R.E.W., Rozek A. (2002). Role of membranes in the activities of antimicrobial cationic peptides. FEMS Microbiol. Lett..

[10] Brown K.L., Hancock R.E.W. (2006). Cationic host defense (antimicrobial) peptides. Curr. Opin. Immunol..

[11] Mangoni M.L., Marcellini H.G.L., Simmaco M. (2007). Biological characterization and modes of action of temporins and bombinins H, multiple forms of short and mildly cationic anti-microbial peptides from amphibian skin. J. Pept. Sci..

[12] Harris F., Dennison S.R., Phoenix D.A. (2009). Anionic antimicrobial peptides from eukaryotic organisms. Curr. Protein Pept. Sci..

[13] Lai R., Liu H., Hui Lee W., Zhang Y. (2002). An anionic antimicrobial peptide from toad *Bombina maxima*. Biochem. Biophys. Res. Commun..

[14] Steffen H., Rieg S., Wiedemann I., Kalbacher H., Deeg M., Sahl H.-G., Peschel A., Götz F., Garbe C., Schittek B. (2006). Naturally processed dermcidin-derived peptides do not permeabilize bacterial membranes and kill microorganisms irrespective of their charge. Antimicrob. Agents Chemother..

[15] Kragol G., Hoffmann R., Chattergoon M.A., Lovas S., Cudic M., Bulet P., Condie B.A., Rosengren K.J., Montaner L.J., Otvos L. (2002). Identification of crucial residues for the antibacterial activity of the proline-rich peptide, pyrrhocoricin. Eur. J. Biochem..

[16] Chan D.I., Prenner E.J., Vogel H.J. (2006). Tryptophan- and arginine-rich antimicrobial peptides: Structures and mechanisms of action. Biochim. Biophys. Acta Biomembr..

[17] Dong N., Ma Q., Shan A., Lv Y., Hu W., Gu Y., Li Y. (2012). Strand length-dependent antimicrobial activity and membrane-active mechanism of arginine- and valine-rich β-hairpin-like antimicrobial peptides. Antimicrob. Agents Chemother..

[18] Baumann T., Kämpfer U., Schürch S., Schaller J., Largiadèr C., Nentwig W., Kuhn-Nentwig L. (2010). Ctenidins: Antimicrobial glycine-rich peptides from the hemocytes of the spider *Cupiennius salei*. Cell. Mol. Life Sci..

[19] Ilić N., Novković M., Guida F., Xhindoli D., Benincasa M., Tossi A., Juretić D. (2013). Selective antimicrobial activity and mode of action of adepantins, glycine-rich peptide antibiotics based on anuran antimicrobial peptide sequences. Biochim. Biophys. Acta Biomembr..

[20] Selsted M.E., Brown D.M., DeLange R.J., Harwig S.S., Lehrer R.I. (1985). Primary structures of six antimicrobial peptides of rabbit peritoneal neutrophils. J. Biol. Chem..

[21] Oppenheim F.G., Xu T., McMillian F.M., Levitz S.M., Diamond R.D., Offner G.D., Troxler R.F. (1988). Histatins, a novel family of histidine-rich proteins in human parotid secretion. Isolation, characterization, primary structure, and fungistatic effects on *Candida albicans*. J. Biol. Chem..

[22] Zasloff M. (2002). Antimicrobial peptides of multicellular organisms. Nature.

[23] Nguyen L.T., Haney E.F., Vogel H.J. (2011). The expanding scope of antimicrobial peptide structures and their modes of action. Trends Biotechnol..

[24] Zasloff M. (1987). Magainins, a class of antimicrobial peptides from Xenopus skin: Isolation, characterization of two active forms, and partial cDNA sequence of a precursor. Proc. Natl. Acad. Sci. USA.

[25] Fahrner R.L., Dieckmann T., Harwig S.S.L., Lehrer R.I., Eisenberg D., Feigon J. (1996). Solution structure of protegrin-1, a broad-spectrum antimicrobial peptide from porcine leukocytes. Chem. Biol..

[26] Scott M.G., Davidson D.J., Gold M.R., Bowdish D., Hancock R.E.W. (2002). The human antimicrobial peptide ll-37 is a multifunctional modulator of innate immune responses. J. Immunol..

[27] Bulet P., Stöcklin R., Menin L. (2004). Anti-microbial peptides: From invertebrates to vertebrates. Immunol. Rev..

[28] Selsted M.E., Harwig S.S., Ganz T., Schilling J.W., Lehrer R.I. (1985). Primary structures of three human neutrophil defensins. J. Clin. Investig..

[29] Fehlbaum P., Bulet P., Michaut L., Lagueux M., Broekaert W.F., Hetru C., Hoffmann J.A. (1994). Insect immunity. Septic injury of Drosophila induces the synthesis of a potent antifungal peptide with sequence homology to plant antifungal peptides. J. Biol. Chem..

[30] Stec B. (2006). Plant thionins—The structural perspective. Cell. Mol. Life Sci..

[31] Mygind P.H., Fischer R.L., Schnorr K.M., Hansen M.T., Sonksen C.P., Ludvigsen S., Raventos D., Buskov S., Christensen B., De Maria L. (2005). Plectasin is a peptide antibiotic with therapeutic potential from a saprophytic fungus. Nature.

[32] Casteels P., Ampe C., Jacobs F., Vaeck M., Tempst P. (1989). Apidaecins: Antibacterial peptides from honeybees. EMBO J..

[33] Cociancich S., Dupont A., Hegy G., Lanot R., Holder F., Hetru C., Hoffmann J.A., Bulet P. (1994). Novel inducible antibacterial peptides from a hemipteran insect, the sap-sucking bug *Pyrrhocoris apterus*. Biochem. J..

[34] Selsted M.E., Novotny M.J., Morris W.L., Tang Y.Q., Smith W., Cullor J.S. (1992). Indolicidin, a novel bactericidal tridecapeptide amide from neutrophils. J. Biol. Chem..

[35] Lawyer C., Pai S., Watabe M., Borgia P., Mashimo T., Eagleton L., Watabe K. (1996). Antimicrobial activity of a 13 amino acid tryptophan-rich peptide derived from a putative porcine precursor protein of a novel family of antibacterial peptides. FEBS Lett..

[36] Phillips R.L., Palombo E.A., Panozzo J.F., Bhave M. (2011). Puroindolines, Pin alleles, hordoindolines and grain softness proteins are sources of bactericidal and fungicidal peptides. J. Cereal Sci..

[37] Hsu S.-T.D., Breukink E., Tischenko E., Lutters M.A.G., de Kruijff B., Kaptein R., Bonvin A.M.J.J., van Nuland N.A.J. (2004). The nisin-lipid II complex reveals a pyrophosphate cage that provides a blueprint for novel antibiotics. Nat. Struct. Mol. Biol..

[38] Véronique C., Birgitta A., Jan J., Fabrice H., Erik G., Jean-Marie R. (2005). Secondary structure and membrane interaction of PR-39, a Pro+Arg-rich antibacterial peptide. Eur. J. Biochem..

[39] Gennaro R., Zanetti M., Benincasa M., Podda E., Miani M. (2002). Pro-rich antimicrobial peptides from animals: Structure, biological functions and mechanism of action. Curr. Pharm. Des..

[40] Bellamy W., Takase M., Wakabayashi H., Kawase K., Tomita M. (1992). Antibacterial spectrum of lactoferricin B, a potent bactericidal peptide derived from the N-terminal region of bovine lactoferrin. J. Appl. Bacteriol..

[41] Lehrer R.I., Cole A.M., Selsted M.E. (2012). θ-Defensins: Cyclic Peptides with Endless Potential. J. Biol. Chem..

[42] Cotter P.D., Ross R.P., Hill C. (2012). Bacteriocins—A viable alternative to antibiotics?. Nat. Rev. Microbiol..

[43] Scheinpflug K., Krylova O., Nikolenko H., Thurm C., Dathe M. (2015). Evidence for a novel mechanism of antimicrobial action of a cyclic R-,W-rich hexapeptide. PLoS ONE.

[44] Craik D.J., Daly N.L., Bond T., Waine C. (1999). Plant cyclotides: A unique family of cyclic and knotted proteins that defines the cyclic cystine knot structural motif1 1 Edited by P. E. Wright. J. Mol. Biol..

[45] Lee D.L., Hodges R.S. (2003). Structure–activity relationships of de novo designed cyclic antimicrobial peptides based on gramicidin S. Pept. Sci..

[46] Rabanal F., Cajal Y. (2017). Recent advances and perspectives in the design and development of polymyxins. Nat. Prod. Rep..

[47] Cirac D.A., Torné M., Badosa E., Montesinos E., Salvador P., Feliu L., Planas M. (2017). Rational design of cyclic antimicrobial peptides based on BPC194 and BPC198. Molecules.

[48] Mishra K.A., Choi J., Choi S.-J., Baek K.-H. (2017). Cyclodipeptides: An Overview of Their Biosynthesis and Biological Activity. Molecules.

[49] Zorzi A., Deyle K., Heinis C. (2017). Cyclic peptide therapeutics: Past, present and future. Curr. Opin. Chem. Biol..

[50] Wang G., Li X., Wang Z. (2016). APD3: The antimicrobial peptide database as a tool for research and education. Nucleic Acids Res..

[51] Rabanal F., Cajal Y., Villa T.G., Vinas M. (2016). Therapeutic potential of antimicrobial peptides. New Weapons to Control Bacterial Growth.

[52] Schauber J., Gallo R.L. (2008). Antimicrobial peptides and the skin immune defense system. J. Allergy Clin. Immunol..

[53] Hilchie A.L., Wuerth K., Hancock R.E.W. (2013). Immune modulation by multifaceted cationic host defense (antimicrobial) peptides. Nat. Chem. Biol..

[54] Conlon J.M., Mechkarska M. (2014). Host-defense peptides with therapeutic potential from skin secretions of frogs from the family pipidae. Pharmaceuticals.

[55] Mylonakis E., Podsiadlowski L., Muhammed M., Vilcinskas A. (2016). Diversity, evolution and medical applications of insect antimicrobial peptides. Philos. Trans. R. Soc. B Biol. Sci..

[56] Zanjani N.T., Miranda-Saksena M., Cunningham A.L., Dehghani F. (2017). Antimicrobial peptides of marine crustaceans: The potential and challenges of developing therapeutic agents. Curr. Med. Chem..

[57] Tam J.P., Wang S., Wong K.H., Tan W.L. (2015). Antimicrobial peptides from plants. Pharmaceuticals.

[58] Wang G. (2014). Human antimicrobial peptides and proteins. Pharmaceuticals.

[59] Dutta P.D., Das S. (2016). Mammalian antimicrobial peptides: Promising therapeutic targets against infection and chronic inflammation. Curr. Top. Med. Chem..

[60] Hassan M., Kjos M., Nes I.F., Diep D.B., Lotfipour F. (2012). Natural antimicrobial peptides from bacteria: Characteristics and potential applications to fight against antibiotic resistance. J. Appl. Microbiol..

[61] Matejuk A., Leng Q., Begum M.D., Woodle M.C., Scaria P., Chou S.-T., Mixson A.J. (2010). Peptide-based antifungal therapies against emerging infections. Drugs Future.

[62] Masso-Silva J.A., Diamond G. (2014). Antimicrobial peptides from fish. Pharmaceuticals.

[63] Li C., Blencke H.-M., Haug T., Stensvåg K. (2015). Antimicrobial peptides in echinoderm host defense. Dev. Comp. Immunol..

[64] Pasupuleti M., Schmidtchen A., Malmsten M. (2012). Antimicrobial peptides: Key components of the innate immune system. Crit. Rev. Biotechnol..

[65] Steiner H. (1982). Secondary structure of the cecropins: Antibacterial peptides from the moth *Hyalophora cecropia*. FEBS Lett..

[66] Shin S.Y., Kang J.H., Lee M.K., Kim S.Y., Kim Y., Hahm K.-S. (1998). Cecropin A—Magainin 2 hybrid peptides having potent antimicrobial activity with low hemolytic effect. IUBMB Life.

[67] Bechinger B., Zasloff M., Opella S.J. (1993). Structure and orientation of the antibiotic peptide magainin in membranes by solid-state nuclear magnetic resonance spectroscopy. Protein Sci..

[68] Selsted M.E., Ouellette A.J. (2005). Mammalian defensins in the antimicrobial immune response. Nat. Immunol..

[69] Falla T.J., Karunaratne D.N., Hancock R.E.W. (1996). Mode of action of the antimicrobial peptide indolicidin. J. Biol. Chem..

[70] Shi J., Ross C.R., Chengappa M.M., Sylte M.J., McVey D.S., Blecha F. (1996). Antibacterial activity of a synthetic peptide (PR-26) derived from PR-39, a proline-arginine-rich neutrophil antimicrobial peptide. Antimicrob. Agents Chemother..

[71] Kościuczuk E.M., Lisowski P., Jarczak J., Strzałkowska N., Jóźwik A., Horbańczuk J., Krzyżewski J., Zwierzchowski L., Bagnicka E. (2012). Cathelicidins: Family of antimicrobial peptides. A review. Mol. Biol. Rep..

[72] Gennaro R., Zanetti M. (2000). Structural features and biological activities of the cathelicidin-derived antimicrobial peptides. Biopolym. Pept. Sci. Sect..

[73] Bals R., Wilson J.M. (2003). Cathelicidins—A family of multifunctional antimicrobial peptides. Cell. Mol. Life Sci..

[74] Shinnar A.E., Butler K.L., Park H.J. (2003). Cathelicidin family of antimicrobial peptides: Proteolytic processing and protease resistance. Bioorg. Chem..

[75] Cole A.M., Ganz T. (2000). Human antimicrobial peptides: Analysis and application. Biotechniques.

[76] Turner J., Cho Y., Dinh N.-N., Waring A.J., Lehrer R.I. (1998). Activities of LL-37, a Cathelin-associated antimicrobial peptide of human neutrophils. Antimicrob. Agents Chemother..

[77] Lee C.-C., Sun Y., Qian S., Huang H.W. (2011). Transmembrane pores formed by human antimicrobial peptide LL-37. Biophys. J..

[78] Kosikowska P., Lesner A. (2016). Antimicrobial peptides (AMPs) as drug candidates: A patent review (2003–2015). Expert Opin. Ther. Patents.

[79] Jenssen H., Hamill P., Hancock R.E.W. (2006). Peptide antimicrobial agents. Clin. Microbiol. Rev..

[80] Yang D., Biragyn A., Kwak L.W., Oppenheim J.J. (2002). Mammalian defensins in immunity: More than just microbicidal. Trends Immunol..

[81] Trabi M., Schirra H.J., Craik D.J. (2001). Three-dimensional structure of RTD-1, a cyclic antimicrobial defensin from *Rhesus Macaque* leukocytes. Biochemistry.

[82] Park C.B., Yi K.-S., Matsuzaki K., Kim M.S., Kim S.C. (2000). Structure–activity analysis of buforin II, a histone H2A-derived antimicrobial peptide: The proline hinge is responsible for the cell-penetrating ability of buforin II. Proc. Natl. Acad. Sci. USA.

[83] Gifford J.L., Hunter H.N., Vogel H.J. (2005). Lactoferricin. Cell. Mol. Life Sci..

[84] Boman H.G. (2000). Innate immunity and the normal microflora. Immunol. Rev..

[85] Boman H.G. (2003). Antibacterial peptides: Basic facts and emerging concepts. J. Intern. Med..

[86] Fernandez-Lopez S., Kim H.-S., Choi E.C., Delgado M., Granja J.R., Khasanov A., Kraehenbuehl K., Long G., Weinberger D.A., Wilcoxen K.M. (2001). Antibacterial agents based on the cyclic d,l-[alpha]-peptide architecture. Nature.

[87] Ge Y., MacDonald D.L., Holroyd K.J., Thornsberry C., Wexler H., Zasloff M. (1999). In vitro antibacterial properties of pexiganan, an analog of magainin. Antimicrob. Agents Chemother..

[88] Rubinchik E., Dugourd D., Algara T., Pasetka C., Friedland H.D. (2009). Antimicrobial and antifungal activities of a novel cationic antimicrobial peptide, omiganan, in experimental skin colonisation models. Int. J. Antimicrob. Agents.

[89] Fjell C.D., Hiss J.A., Hancock R.E.W., Schneider G. (2012). Designing antimicrobial peptides: Form follows function. Nat. Rev. Drug Discov..

[90] Matsuzaki K. (1999). Why and how are peptide–lipid interactions utilized for self-defense? Magainins and tachyplesins as archetypes. Biochim. Biophys. Acta Biomembr..

[91] Breukink E., de Kruijff B. (2006). Lipid II as a target for antibiotics. Nat. Rev. Drug Discov..

[92] Brumfitt W., Salton M.R.J., Hamilton-Miller J.M.T. (2002). Nisin, alone and combined with peptidoglycan-modulating antibiotics: Activity against methicillin-resistant *Staphylococcus aureus* and vancomycin-resistant enterococci. J. Antimicrob. Chemother..

[93] Shai Y. (1999). Mechanism of the binding, insertion and destabilization of phospholipid bilayer membranes by α-helical antimicrobial and cell non-selective membrane-lytic peptides. Biochim. Biophys. Acta Biomembr..

[94] Brogden K.A. (2005). Antimicrobial peptides: Pore formers or metabolic inhibitors in bacteria?. Nat. Rev. Micro..

[95] Hale J.D.F., Hancock R.E.W. (2007). Alternative mechanisms of action of cationic antimicrobial peptides on bacteria. Expert Rev. Anti-infect. Ther..

[96] Yang L., Harroun T.A., Weiss T.M., Ding L., Huang H.W. (2001). Barrel-stave model or toroidal model? A case study on melittin pores. Biophys. J..

[97] Hallock K.J., Lee D.-K., Ramamoorthy A. (2003). MSI-78, an analogue of the magainin antimicrobial peptides, disrupts lipid bilayer structure via positive curvature strain. Biophys. J..

[98] Yoneyama F., Imura Y., Ohno K., Zendo T., Nakayama J., Matsuzaki K., Sonomoto K. (2009). Peptide-lipid huge toroidal pore, a new antimicrobial mechanism mediated by a lactococcal bacteriocin, lacticin Q. Antimicrob. Agents Chemother..

[99] Gazit E., Boman A., Boman H.G., Shai Y. (1995). Interaction of the Mammalian antibacterial peptide cecropin P1 with phospholipid vesicles. Biochemistry.

[100] Bahar A.A., Ren D. (2013). Antimicrobial peptides. Pharmaceuticals.

[101] Subbalakshmi C., Sitaram N. (1998). Mechanism of antimicrobial action of indolicidin. FEMS Microbiol. Lett..

[102] Otvos L., Rogers M.E., Consolvo P.J., Condie B.A., Lovas S., Bulet P., Blaszczyk-Thurin M. (2000). Interaction between heat shock proteins and antimicrobial peptides. Biochemistry.

[103] Brötz H., Bierbaum G., Leopold K., Reynolds P.E., Sahl H.-G. (1998). The lantibiotic mersacidin inhibits peptidoglycan synthesis by targeting Lipid II. Antimicrob. Agents Chemother..

[104] Park C.B., Kim H.S., Kim S.C. (1998). Mechanism of action of the antimicrobial peptide buforin II: Buforin II kills microorganisms by penetrating the cell membrane and inhibiting cellular functions. Biochem. Biophys. Res. Commun..

[105] Krizsan A., Volke D., Weinert S., Sträter N., Knappe D., Hoffmann R. (2014). Insect-derived proline-rich antimicrobial peptides kill bacteria by inhibiting bacterial protein translation at the 70 S ribosome. Angew. Chem. Int. Ed..

[106] Graf M., Mardirossian M., Nguyen F., Seefeldt A.C., Guichard G., Scocchi M., Innis C.A., Wilson D.N. (2017). Proline-rich antimicrobial peptides targeting protein synthesis. Nat. Prod. Rep..

[107] Madani F., Lindberg S., Langel Ü., Futaki S., Gräslund A. (2011). Mechanisms of cellular uptake of cell-penetrating peptides. J. Biophys..

[108] Jones A.T. (2007). Macropinocytosis: Searching for an endocytic identity and role in the uptake of cell penetrating peptides. J. Cell. Mol. Med..

[109] Mayor S., Pagano R.E. (2007). Pathways of clathrin-independent endocytosis. Nat. Rev. Mol. Cell Biol..

[110] Gordon Y.J., Romanowski E.G., McDermott A.M. (2005). A Review of antimicrobial peptides and their therapeutic potential as anti-infective drugs. Curr. Eye Res..

[111] Blondelle S.E., Pérez-Payá E., Houghten R.A. (1996). Synthetic combinatorial libraries: Novel discovery strategy for identification of antimicrobial agents. Antimicrob. Agents Chemother..

[112] Fields G.B., Noble R.L. (1990). Solid phase peptide synthesis utilizing 9-fluorenylmethoxycarbonyl amino acids. Int. J. Pept. Protein Res..

[113] Houghten R.A., Pinilla C., Blondelle S.E., Appel J.R., Dooley C.T., Cuervo J.H. (1991). Generation and use of synthetic peptide combinatorial libraries for basic research and drug discovery. Nature.

[114] Houghten R.A., Appel J.R., Blondelle S.E., Cuervo J.H., Dooley C.T., Pinilla C. (1992). The use of synthetic peptide combinatorial libraries for the identification of bioactive peptides. Biotechniques.

[115] Houghten R.A., Pinilla C., Appel J.R., Blondelle S.E., Dooley C.T., Eichler J., Nefzi A., Ostresh J.M. (1999). Mixture-based synthetic combinatorial libraries. J. Med. Chem..

[116] Blondelle S.E., Takahashi E., Houghten R.A., Pérez-Payá E. (1996). Rapid identification of compounds with enhanced antimicrobial activity by using conformationally defined combinatorial libraries. Biochem. J..

[117] López-García B., Pérez-Payá E., Marcos J.F. (2002). Identification of novel hexapeptides bioactive against phytopathogenic fungi through screening of a synthetic peptide combinatorial library. Appl. Environ. Microbiol..

[118] Hong S.Y., Oh J.E., yun Kwon M., Choi M.J., Lee J.H., Lee B.L., Moon H.M., Lee K.H. (1998). Identification and characterization of novel antimicrobial decapeptides generated by combinatorial chemistry. Antimicrob. Agents Chemother..

[119] Strøm M.B., Haug B.E., Skar M.L., Stensen W., Stiberg T., Svendsen J.S. (2003). The pharmacophore of short cationic antibacterial peptides. J. Med. Chem..

[120] Liu Z., Brady A., Young A., Rasimick B., Chen K., Zhou C., Kallenbach N.R. (2007). Length effects in antimicrobial peptides of the (RW)_n_ Series. Antimicrob. Agents Chemother..

[121] Blondelle S.E., Lohner K. (2000). Combinatorial libraries: A tool to design antimicrobial and antifungal peptide analogues having lytic specificities for structure-activity relationship studies. Biopolymers.

[122] Choi J., Moon E. (2009). Identification of novel bioactive hexapeptides against phytopathogenic bacteria through rapid screening of a synthetic combinatorial library. J. Microbiol. Biotechnol..

[123] Choi J., Park E., Lee S.W., Hyun J.W., Baek K.H. (2017). Selection of small synthetic antimicrobial peptides inhibiting *Xanthomonas citri* subsp. *citri* causing citrus canker. Plant Pathol. J..

[124] Blondelle S.E., Takahashi E., Weber P.A., Houghten R.A. (1994). Identification of antimicrobial peptides by using combinatorial libraries made up of unnatural amino acids. Antimicrob. Agents Chemother..

[125] Monroc S., Badosa E., Feliu L., Planas M., Montesinos E., Bardají E. (2006). De novo designed cyclic cationic peptides as inhibitors of plant pathogenic bacteria. Peptides.

[126] Monroc S., Badosa E., Besalú E., Planas M., Bardají E., Montesinos E., Feliu L. (2006). Improvement of cyclic decapeptides against plant pathogenic bacteria using a combinatorial chemistry approach. Peptides.

[127] Shagaghi N., Palombo E.A., Clayton A.H.A., Bhave M. (2016). Archetypal tryptophan-rich antimicrobial peptides: Properties and applications. World J. Microbiol. Biotechnol..

[128] Schibli D.J., Epand R.F., Vogel H.J., Epand R.M. (2002). Tryptophan-rich antimicrobial peptides: Comparative properties and membrane interactions. Biochem. Cell Biol..

[129] Trinquier G., Sanejouand Y.H. (1998). Which effective property of amino acids is best preserved by the genetic code?. Protein Eng. Des. Sel..

[130] Strøm M.B., Svendsen J.S., Rekdal Ø. (2000). Antibacterial activity of 15-residue lactoferricin derivatives. J. Pept. Res..

[131] Yau W.-M., Wimley W.C., Gawrisch K., White S.H. (1998). The preference of tryptophan for membrane interfaces. Biochemistry.

[132] Khandelia H., Kaznessis Y.N. (2007). Cation–π interactions stabilize the structure of the antimicrobial peptide indolicidin near membranes: Molecular dynamics simulations. J. Phys. Chem. B.

[133] Wessolowski A., Bienert M., Dathe M. (2004). Antimicrobial activity of arginine- and tryptophan-rich hexapeptides: The effects of aromatic clusters, d-amino acid substitution and cyclization. J. Pept. Res..

[134] Haug B.E., Skar M.L., Svendsen J.S. (2001). Bulky aromatic amino acids increase the antibacterial activity of 15-residue bovine lactoferricin derivatives. J. Pept. Sci..

[135] Schiffer M., Chang C.-H., Stevens F.J. (1992). The function of tryptophan residues in membrane proteins. Protein Eng. Des. Sel..

[136] Strøm M.B., Rekdal Ø., Svendsen J.S. (2002). Antimicrobial activity of short arginine- and tryptophan-rich peptides. J. Pept. Sci..

[137] Strom M., Hansen T., Havelkova M., Torfoss V. (2014). Therapeutic Peptides.

[138] Bi X., Wang C., Ma L., Sun Y., Shang D. (2013). Investigation of the role of tryptophan residues in cationic antimicrobial peptides to determine the mechanism of antimicrobial action. J. Appl. Microbiol..

[139] Arias M., Nguyen L.T., Kuczynski A.M., Lejon T., Vogel H.J. (2014). Position-dependent influence of the three trp residues on the membrane activity of the antimicrobial peptide, tritrpticin. Antibiotics.

[140] Reed J.D., Edwards D.L., Gonzalez C.F. (1997). Synthetic peptide combinatorial libraries: A method for the identification of bioactive peptides against phytopathogenic fungi. Mol. Plant-Microbe Interact..

[141] Tomita M., Takase M., Bellamy W., Shimamura S. (1994). A review: The active peptide of lactoferrin. Pediatr. Int..

[142] Robinson W.E., McDougall B., Tran D., Selsted M.E. (1998). Anti-HIV-1 activity of indolicidin, an antimicrobial peptide from neutrophils. J. Leukoc. Biol..

[143] Albiol Matanic V.C., Castilla V. (2004). Antiviral activity of antimicrobial cationic peptides against Junin virus and herpes simplex virus. Int. J. Antimicrob. Agents.

[144] Bruni N., Capucchio T.M., Biasibetti E., Pessione E., Cirrincione S., Giraudo L., Corona A., Dosio F. (2016). Antimicrobial activity of lactoferrin-related peptides and applications in human and veterinary medicine. Molecules.

[145] Hasegawa K., Motsuchi W., Tanaka S., Dosako S. (1994). Inhibition with lactoferrin of in vitro infection with human herpes virus. Jpn. J. Med. Sci. Biol..

[146] Ikeda M., Sugiyama K., Tanaka T., Tanaka K., Sekihara H., Shimotohno K., Kato N. (1998). Lactoferrin markedly inhibits hepatitis C virus infection in cultured human hepatocytes. Biochem. Biophys. Res. Commun..

[147] Cirioni O., Giacometti A., Silvestri C., Della Vittoria A., Licci A., Riva A., Scalise G. (2006). In vitro activities of tritrpticin alone and in combination with other antimicrobial agents against *Pseudomonas aeruginosa*. Antimicrob. Agents Chemother..

[148] Infante V.V., Miranda-Olvera A.D., De Leon-Rodriguez L.M., Anaya-Velazquez F., Rodriguez M.C., Avila E.E. (2011). Effect of the antimicrobial peptide tritrpticin on the in vitro viability and growth of *Trichomonas vaginalis*. Curr. Microbiol..

[149] Yang S.-T., Yub Shin S., Kim Y.-C., Kim Y., Hahm K.-S., Kim J.I. (2002). Conformation-dependent antibiotic activity of tritrpticin, a cathelicidin-derived antimicrobial peptide. Biochem. Biophys. Res. Commun..

[150] Jing W., Demcoe A.R., Vogel H.J. (2003). Conformation of a bactericidal domain of puroindoline a: Structure and mechanism of action of a 13-residue antimicrobial peptide. J. Bacteriol..

[151] Alfred R.L., Palombo E.A., Panozzo J.F., Bariana H., Bhave M. (2013). Stability of puroindoline peptides and effects on wheat rust. World J. Microbiol. Biotechnol..

[152] Choi J., Baek K.H., Moon E. (2014). Antimicrobial effects of a hexapetide KCM21 against *Pseudomonas syringae* pv. tomato DC3000 and *Clavibacter michiganensis* subsp. michiganensis. Plant Pathol. J..

[153] Wakabayashi H., Hiratani T., Uchida K., Yamaguchi H. (1996). Antifungal spectrum and fungicidal mechanism of an n-terminal peptide of bovine lactoferrin. J. Infect. Chemother..

[154] Sengupta J., Saha S., Khetan A., Sarkar S.K., Mandal S.M. (2012). Effects of lactoferricin B against keratitis-associated fungal biofilms. J. Infect. Chemother..

[155] Pellegrini A., Thomas U., Bramaz N., Klauser S., Hunziker P., Von Fellenberg R. (1997). Identification and isolation of a bactericidal domain in chicken egg white lysozyme. J. Appl. Microbiol..

[156] Gopal R., Na H., Seo C.H., Park Y. (2012). Antifungal activity of (KW)_n_ or (RW)_n_ Peptide against *Fusarium solani* and *Fusarium oxysporum*. Int. J. Mol. Sci..

[157] Won H.-S., Kang S.-J., Choi W.-S., Lee B.-J. (2011). Activity optimization of an undecapeptide analogue derived from a frog-skin antimicrobial peptide. Mol. Cells.

[158] Da Silva A., Kawazoe U., Freitas F.F.T., Gatti M.S.V., Dolder H., Schumacher R.I., Juliano M.A., da Silva M.J., Leite A. (2002). Avian anticoccidial activity of a novel membrane-interactive peptide selected from phage display libraries. Mol. Biochem. Parasitol..

[159] Scocchi M., Tossi A., Gennaro R. (2011). Proline-rich antimicrobial peptides: Converging to a non-lytic mechanism of action. Cell. Mol. Life Sci..

[160] Li W., Tailhades J., O’Brien-Simpson N.M., Separovic F., Otvos L., Hossain M.A., Wade J.D. (2014). Proline-rich antimicrobial peptides: Potential therapeutics against antibiotic-resistant bacteria. Amino Acids.

[161] Vitali A. (2015). Proline-rich peptides: Multifunctional bioactive molecules as new potential therapeutic drugs. Curr. Protein Pept. Sci..

[162] Fang W.-Y., Dahiya R., Qin H.-L., Mourya R., Maharaj S. (2016). Natural proline-rich cyclopolypeptides from marine organisms: Chemistry, synthetic methodologies and biological status. Mar. Drugs.

[163] Runti G., Lopez Ruiz M.D.C., Stoilova T., Hussain R., Jennions M., Choudhury H.G., Benincasa M., Gennaro R., Beis K., Scocchi M. (2013). Functional characterization of SbmA, a bacterial inner membrane transporter required for importing the antimicrobial peptide Bac7(1-35). J. Bacteriol..

[164] Mattiuzzo M., Bandiera A., Gennaro R., Benincasa M., Pacor S., Antcheva N., Scocchi M. (2007). Role of the *Escherichia coli* SbmA in the antimicrobial activity of proline-rich peptides. Mol. Microbiol..

[165] Kragol G., Lovas S., Varadi G., Condie B.A., Hoffmann R., Otvos L. (2001). The antibacterial peptide pyrrhocoricin inhibits the ATPase actions of DnaK and Prevents chaperone-assisted protein folding. Biochemistry.

[166] Calloni G., Chen T., Schermann S.M., Chang H., Genevaux P., Agostini F., Tartaglia G.G., Hayer-Hartl M., Hartl F.U. (2012). DnaK functions as a central hub in the *E. coli* chaperone network. Cell Rep..

[167] Berthold N., Hoffmann R. (2014). Cellular uptake of apidaecin 1b and related analogs in gram-negative bacteria reveals novel antibacterial mechanism for proline-rich antimicrobial peptides. Protein Pept. Lett..

[168] Gagnon M.G., Roy R.N., Lomakin I.B., Florin T., Mankin A.S., Steitz T.A. (2016). Structures of proline-rich peptides bound to the ribosome reveal a common mechanism of protein synthesis inhibition. Nucleic Acids Res..

[169] Casteels P., Ampe C., Rivière L., DAMME J., Elicone C., Fleming M., Jacobs F., Tempst P. (1990). Isolation and characterization of abaecin, a major antibacterial response peptide in the honeybee (*Apis mellifera*). Eur. J. Biochem..

[170] Bulet P., Dimarcq J.L., Hetru C., Lagueux M., Charlet M., Hegy G., Van Dorsselaer A., Hoffmann J.A. (1993). A novel inducible antibacterial peptide of Drosophila carries an O-glycosylated substitution. J. Biol. Chem..

[171] Chernysh S., Cociancich S., Briand J.-P., Hetru C., Bulet P. (1996). The inducible antibacterial peptides of the Hemipteran insect *Palomena prasina*: Identification of a unique family of prolinerich peptides and of a novel insect defensin. J. Insect Physiol..

[172] Knappe D., Piantavigna S., Hansen A., Mechler A., Binas A., Nolte O., Martin L.L., Hoffmann R. (2010). Oncocin (VDKPPYLPRPRPPRRIYNR-NH_2_): A Novel antibacterial peptide optimized against gram-negative human pathogens. J. Med. Chem..

[173] Stensvåg K., Haug T., Sperstad S.V., Rekdal Ø., Indrevoll B., Styrvold O.B. (2008). Arasin 1, a proline–arginine-rich antimicrobial peptide isolated from the spider crab, *Hyas araneus*. Dev. Comp. Immunol..

[174] Kim J.K., Son D.W., Kim C.-H., Cho J.H., Marchetti R., Silipo A., Sturiale L., Park H.Y., Huh Y.R., Nakayama H. (2015). Insect gut symbiont susceptibility to host antimicrobial peptides caused by alteration of the bacterial cell envelope. J. Biol. Chem..

[175] Park K.-E., Jang S.H., Lee J., Lee S.A., Kikuchi Y., Seo Y., Lee B.L. (2018). The roles of antimicrobial peptide, rip-thanatin, in the midgut of *Riptortus pedestris*. Dev. Comp. Immunol..

[176] Gennaro R., Skerlavaj B., Romeo D. (1989). Purification, composition, and activity of two bactenecins, antibacterial peptides of bovine neutrophils. Infect. Immun..

[177] Agerberth B., Lee J.-Y., Bergman T., Carlquist M., Boman H.G., Mutt V., Jörnvall H. (1991). Amino acid sequence of PR-39. Eur. J. Biochem..

[178] Otvos L. (2002). The short proline-rich antibacterial peptide family. Cell. Mol. Life Sci..

[179] Rahnamaeian M., Langen G., Imani J., Khalifa W., Altincicek B., von Wettstein D., Kogel K.-H., Vilcinskas A. (2009). Insect peptide metchnikowin confers on barley a selective capacity for resistance to fungal ascomycetes pathogens. J. Exp. Bot..

[180] Rahnamaeian M., Vilcinskas A. (2012). Defense gene expression is potentiated in transgenic barley expressing antifungal peptide metchnikowin throughout powdery mildew challenge. J. Plant Res..

[181] Stalmans S., Wynendaele E., Bracke N., Knappe D., Hoffmann R., Peremans K., Polis I., Burvenich C., De Spiegeleer B. (2014). Blood-brain barrier transport of short proline-rich antimicrobial peptides. Protein Pept. Lett..

[182] Lalatsa A., Schatzlein A.G., Uchegbu I.F. (2014). Strategies to deliver peptide drugs to the brain. Mol. Pharm..

[183] Roy R.N., Lomakin I.B., Gagnon M.G., Steitz T.A. (2015). The mechanism of inhibition of protein synthesis by the proline-rich peptide oncocin. Nat. Struct. Mol. Biol..

[184] Destoumieux D., Munoz M., Bulet P., Bachère E. (2000). Penaeidins, a family of antimicrobial peptides from penaeid shrimp (Crustacea, Decapoda). Cell. Mol. Life Sci..

[185] Mardirossian M., Grzela R., Giglione C., Meinnel T., Gennaro R., Mergaert P., Scocchi M. (2014). The host antimicrobial peptide Bac71-35 binds to bacterial ribosomal proteins and inhibits protein synthesis. Chem. Biol..

[186] Runti G., Benincasa M., Giuffrida G., Devescovi G., Venturi V., Gennaro R., Scocchi M. (2017). The mechanism of killing by the proline-rich peptide Bac7(1–35) against clinical strains of pseudomonas aeruginosa differs from that against other gram-negative bacteria. Antimicrob. Agents Chemother..

[187] Tian W., Li B., Zhang X., Dang W., Wang X., Tang H., Wang L., Cao H., Chen T. (2012). Suppression of tumor invasion and migration in breast cancer cells following delivery of siRNA against Stat3 with the antimicrobial peptide PR39. Oncol. Rep..

[188] Cao H., Ke T., Liu R., Yu J., Dong C., Cheng M., Huang J., Liu S. (2015). Identification of a novel proline-rich antimicrobial peptide from *Brassica napus*. PLoS ONE.

[189] Yeaman M.R., Yount N.Y. (2003). Mechanisms of antimicrobial peptide action and resistance. Pharmacol. Rev..

[190] Lewis L.A., Choudhury B., Balthazar J.T., Martin L.E., Ram S., Rice P.A., Stephens D.S., Carlson R., Shafer W.M. (2009). Phosphoethanolamine substitution of lipid A and resistance of Neisseria gonorrhoeae to Cationic Antimicrobial Peptides and Complement-Mediated Killing by Normal Human Serum. Infect. Immun..

[191] Gunn J.S. (2001). Bacterial modification of LPS and resistance to antimicrobial peptides. J. Endotoxin Res..

[192] Guo L., Lim K.B., Poduje C.M., Daniel M., Gunn J.S., Hackett M., Miller S.I. (1998). Lipid A acylation and bacterial resistance against vertebrate antimicrobial peptides. Cell.

[193] Guina T., Yi E.C., Wang H., Hackett M., Miller S.I. (2000). A PhoP-regulated outer membrane protease of *Salmonella enterica* Serovar typhimurium Promotes Resistance to Alpha-helical antimicrobial peptides. J. Bacteriol..

[194] Shafer W.M., Qu X.-D., Waring A.J., Lehrer R.I. (1998). Modulation of *Neisseria gonorrhoeae* susceptibility to vertebrate antibacterial peptides due to a member of the resistance/nodulation/division efflux pump family. Proc. Natl. Acad. Sci. USA.

[195] Del Castillo F.J., del Castillo I., Moreno F. (2001). Construction and characterization of mutations at codon 751 of the *Escherichia coli* gyrB gene that confer resistance to the antimicrobial peptide microcin B17 and alter the activity of DNA gyrase. J. Bacteriol..

[196] Friedrich C., Scott M.G., Karunaratne N., Yan H., Hancock R.E.W. (1999). Salt-resistant alpha-helical cationic antimicrobial peptides. Antimicrob. Agents Chemother..

[197] Yeaman M.R., Bayer A.S., Koo S.P., Foss W., Sullam P.M. (1998). Platelet microbicidal proteins and neutrophil defensin disrupt the *Staphylococcus aureus* cytoplasmic membrane by distinct mechanisms of action. J. Clin. Investig..

[198] Vuong C., Voyich J.M., Fischer E.R., Braughton K.R., Whitney A.R., DeLeo F.R., Otto M. (2004). Polysaccharide intercellular adhesin (PIA) protects *Staphylococcus epidermidis* against major components of the human innate immune system. Cell. Microbiol..

[199] Miller S.I., Kukral A.M., Mekalanos J.J. (1989). A two-component regulatory system (PhoP PhoQ) controls *Salmonella typhimurium* virulence. Proc. Natl. Acad. Sci. USA.

[200] Bader M.W., Sanowar S., Daley M.E., Schneider A.R., Cho U., Xu W., Klevit R.E., Le Moual H., Miller S.I. (2005). Recognition of antimicrobial peptides by a bacterial sensor kinase. Cell.

